# A Vision-Based Single-Sensor Approach for Identification and Localization of Unloading Hoppers

**DOI:** 10.3390/s25144330

**Published:** 2025-07-10

**Authors:** Wuzhen Wang, Tianyu Ji, Qi Xu, Chunyi Su, Guangming Zhang

**Affiliations:** College of Electrical Engineering and Control Science, Nanjing Tech University, Nanjing 211816, China; wangwuzhen@njtech.edu.cn (W.W.); 202361206163@njtech.edu.cn (T.J.); xenon@njtech.edu.cn (Q.X.); chunysu@163.com (C.S.)

**Keywords:** automatic control, object detection, edge detection, 3D localization system

## Abstract

To promote the automation and intelligence of rail freight, the accurate identification and localization of bulk cargo unloading hoppers have become a key technical challenge. Under the technological wave driven by the deep integration of Industry 4.0 and artificial intelligence, the bulk cargo unloading process is undergoing a significant transformation from manual operation to intelligent control. In response to this demand, this paper proposes a vision-based 3D localization system for unloading hoppers, which adopts a single visual sensor architecture and integrates three core modules: object detection, corner extraction, and 3D localization. Firstly, a lightweight hybrid attention mechanism is incorporated into the YOLOv5 network to enable edge deployment and enhance the detection accuracy of unloading hoppers in complex industrial scenarios. Secondly, an image processing approach combining depth consistency constraint (DCC) and geometric structure constraints is designed to achieve sub-pixel level extraction of key corner points. Finally, a real-time 3D localization method is realized by integrating corner-based initialization with an RGB-D SLAM tracking mechanism. Experimental results demonstrate that the proposed system achieves an average localization accuracy of 97.07% under challenging working conditions. This system effectively meets the comprehensive requirements of automation, intelligence, and high precision in railway bulk cargo unloading processes, and exhibits strong engineering practicality and application potential.

## 1. Introduction

Over the past two decades, rail freight has remained a cornerstone of national logistics infrastructure and economic development, particularly in resource-rich and geographically expansive countries such as China [1]. With its high capacity, cost-efficiency, and long-distance transport capability, rail freight plays a pivotal role in ensuring the stable delivery of essential bulk materials across regions. Among various commodities, coal—commonly referred to as “black gold”—continues to dominate in terms of freight volume, serving as a primary fuel source for power generation and industrial production. In this context, the stability and efficiency of coal transportation systems directly impact both energy security and industrial output.

However, despite the high logistical importance of coal transport, the final stage—unloading at bulk terminals—remains heavily reliant on manual operation. In most current settings, workers manually align unloading hoppers with freight wagon discharge ports, often guided by experience rather than precise measurement or intelligent control. This not only limits operational efficiency and throughput but also introduces significant safety risks due to misalignment, collision, or hopper malfunction. Furthermore, labor-intensive processes hinder scalability and are inconsistent with the ongoing push toward intelligent, automated logistics systems under national digital transformation initiatives.

To address the alignment and positioning challenges during the unloading process, various engineering solutions have been proposed. Current industrial practices largely fall into two categories: contact-based localization and multi-sensor non-contact systems. Contact-based approaches typically involve mounting GNSS receivers, IMUs, or other hardware sensors on both freight wagons and unloading hoppers to compute relative poses based on global position coordinates. While technically feasible, these systems often suffer from high cost, complicated installation procedures, frequent maintenance, and poor reliability in harsh operational environments, such as dusty, vibrating, or magnetically noisy conditions common in coal and ore unloading stations. Alternatively, multi-camera vision systems attempt to track key features on both the hopper and the wagon using calibrated 3D vision techniques. Yet, they require careful manual calibration, are prone to occlusion, and demand significant computational resources, making them impractical for many real-world deployments.

Amid the ongoing Industry 4.0 [2] transformation and rapid advancements in artificial intelligence, vision-based sensor localization techniques have emerged as promising alternatives. By leveraging stereo vision combined with deep learning for robust object detection, these approaches offer the potential for real-time, accurate, and low-cost localization of unloading equipment in complex industrial settings. Such solutions can significantly enhance automation levels and operational safety while reducing system complexity.

Motivated by these challenges and opportunities, this paper proposes a vision-based single-sensor framework leveraging YOLOv5 for robust object detection and stereo depth estimation for sub-pixel-level corner localization. Unlike previous multi-sensor systems, our method uses only a ZED 2i stereo camera (Stereolabs, San Francisco, CA, USA) to achieve 3D localization with millimeter-level accuracy. By incorporating lite-hybrid attention module and lightweight RepVGG blocks, the framework balances real-time performance with precision in complex industrial settings. The main contributions are as follows:

1. A novel vision-based single-sensor framework is proposed for the identification and 3D localization of unloading hoppers in railway terminals, relying solely on a stereo camera.
2. A hybrid attention module (lite-hybrid attention, LHA) is introduced into YOLOv5’s C3 block, combining ECA-Net and SimAM with group convolution to enhance feature representation at low computational cost.
3. A structured 3D localization pipeline is constructed by integrating sub-pixel corner extraction, stereo depth fusion, and SLAM-based dynamic tracking, achieving high accuracy without external sensors.

The remainder of this paper is organized as follows: Section 2 reviews related work on object detection and visual localization technologies. Section 3 introduces the equipment and system architecture used in this study. Section 4 presents the proposed methodology in detail. Section 5 reports the experimental results based on real-world data. Finally, Section 6 concludes the paper.

## 2. Related Work

In recent years, vision-based approaches have demonstrated significant potential in the identification and monitoring of railway superstructures. P. Aela et al. [3] emphasized the advantages of visual methods in assessing railway operational stability and maintenance requirements. Q. Y. Ran et al. [4] pointed out that China has adopted diversified strategies to simultaneously promote industrial greening and high-end development. Numerous researchers have shown increasing interest in this field and have proposed a variety of innovative methodologies.

### 2.1. Vision-Based Identification of Industrial Targets

In complex industrial environments, the accurate and rapid identification of unloading hoppers within images is one of the key technologies for achieving automated operations. Inspired by the Canny algorithm [5], S. T. Ge et al. [6] developed a cascaded filter based on morphological processing for real-time shape measurement during hot slab rolling. Benefiting from CNN advances, Wu S. et al. [7] propose a sparsity-aware global channel pruning (SAGCP) method for infrared small-target detection, which uses sparse priors to prune redundant channels effectively. Applied to DNA-Net, it reduces parameters and FLOPs by over 50% while slightly improving IoU. C. Mi et al. [8] proposed an automatic vision system for fast container corner casting detection. This system employed histogram of oriented gradients (HOG) descriptors for image preprocessing and utilized a trained support vector machine (SVM) classifier for component identification. By integrating a symmetry-based mirroring algorithm, the system achieved efficient corner localization, demonstrating the robust potential of vision-based methods in industrial scenarios. Zhang’s camera calibration method [9] is widely used due to its flexibility and simplicity. C. Mi et al. [10], based on the pinhole camera model, applied triangulation combined with machine vision to achieve real-time 3D pose estimation of containers by detecting keyhole center offsets. Inspired by advances in AI, Drobnyi V. et al. [11] propose a method to construct geometric digital twins by expanding empty regions in point clouds, enabling indoor modeling beyond Manhattan-world constraints. Their approach handles complex layouts and outperforms existing methods on S3DIS and TUMCMS datasets. Chen Y. H. [12] addressed underwater image distortion by proposing a novel underwater binocular vision system for abalone size measurement. D. M. Feng et al. [13] developed a vision-based sensor system for remote displacement measurement of structures, characterized by its low cost, ease of use, and operational flexibility. However, these methods are not well suited to scenarios involving restricted fields of view and camera motion, limiting their applicability in dynamic industrial environments.

### 2.2. Advances in Object Detection Models

Among object detection models, Xiao C. et al. [14] design a sparse anchor-free detector by converting dense multi-frame inputs into sparse spatio-temporal point clouds, effectively reducing redundant background computation. This method achieves 98.8 FPS on 1024 × 1024 images and reaches state-of-the-art accuracy. Du S.J. et al. [15] proposed an improved YOLOv8s-based method for traffic sign detection by integrating a Space-to-Depth module, Select Kernel attention, and WIoUv3 loss, achieving 3.2% and 5.1% mAP50 improvements on CCTSDB and TT100K datasets, respectively. Liang T.J. et al. [16] enhanced Sparse R-CNN with coordinate attention and feature pyramids, and introduced SAA and DTA modules to boost detection accuracy and robustness in real traffic scenes.

YOLOv5 has emerged as one of the most widely used algorithms in industrial applications due to its high detection speed, scalability, and adaptability to edge deployment environments. In recent years, ongoing research has focused on enhancing the detection accuracy of YOLOv5. For example, Y. L. Zhao et al. [17] introduced a lightweight FF-Slim-Neck module and a parameter-free spatial attention (PSA) mechanism into YOLOv5, which improved detection accuracy but increased model size by 21.6%, negatively impacting deployment on edge devices. Similarly, Y. J. Fang et al. [18] enhanced model robustness and accuracy in complex industrial settings by integrating a CBiFPN feature fusion structure, a C3_CA attention module, and an EDH edge detection head. However, this solution significantly increased model complexity due to the inclusion of multiple components. These studies highlight an ongoing challenge in YOLOv5 optimization: how to maintain detection accuracy while effectively controlling model size for real-time edge applications.

### 2.3. Current Approaches to Industrial Target Localization

Multimodal sensor fusion is also widely adopted in the field. D. Ekaso et al. [19] reported that GNSS RTK mounted on drones introduced up to 0.28 s of delay relative to image acquisition, which significantly compromised 3D localization performance—an issue particularly critical in multimodal sensor fusion applications. Maritime ports and rail freight systems share similar operating targets and unloading procedures. J. Benkert et al. [20] proposed transforming laser-based container crane systems into fully vision-based automation systems capable of integrating both dynamic and static real-time measurements, framing this transformation as both a future challenge and opportunity. Multimodal sensor fusion remains a common approach. Shen Z. Y. et al. [21] proposed a monocular-vision-based hopper localization system that combined GPS and PnP algorithms to localize port equipment, achieving a mean coordinate accuracy exceeding 93%. C. H. Ngo et al. [22] integrated LiDAR and vision sensors to support grab-type unloaders in hold detection, operation point extraction, and collision risk prediction, with errors controlled within a 5–10% range.

## 3. System Overview

### 3.1. Laboratory-Scale Experimental Platform

**Figure 1:**
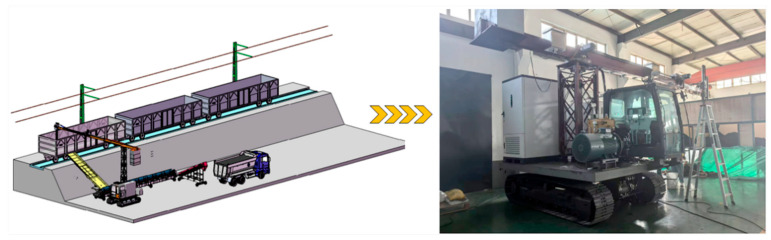
Schematic of the laboratory-scale railway bulk cargo unloading system. Comprising a programmable logic controller (PLC)-driven unloader and a standardized freight wagon. This setup replicates the real-world unloading process for performance evaluation and algorithm validation.

The laboratory-scale railway bulk cargo unloading system consists of an intelligent unloader and a standardized freight wagon, as illustrated in Figure 1. The intelligent unloader is equipped with a programmable logic controller (PLC) and achieves real-time wireless data transmission with the computing terminal via the Snap7 communication protocol. The PLC directly controls the operation of the unloading hopper, and a feedback mechanism is incorporated to establish a closed-loop control system, ensuring continuous, efficient, and automated operation of the entire system.

The simulated freight wagon is constructed at a 1:1 scale, replicating the dimensions of a real railway freight car (13.43 m × 2.892 m × 2.05 m), and is filled with coal as a representative bulk cargo. The system faithfully reproduces the structural layout and functional design of actual industrial scenarios, thereby enabling accurate simulation of the real railway unloading process and providing a reliable platform for related research and technology validation.

### 3.2. System Architecture and Design

This applied study was initiated based on an in-depth investigation of real-world operating sites. The ZED 2i stereo camera was selected as the core visual sensor of the system. Its main technical specifications are listed in Table 1.

**Figure 2:**
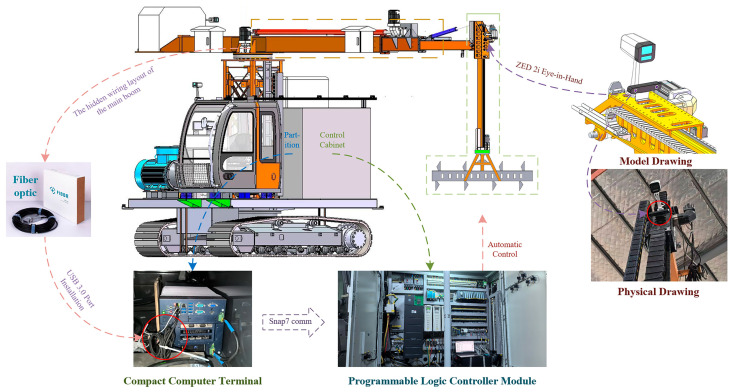
Structural schematic of the bulk cargo unloading system showing the connectivity of the ZED 2i stereo camera, fiber-optic communication, and the high-performance computing terminal used for real-time data processing.

The sensor’s connectivity, shown in Figure 2, adopts fiber-optic communication technology, utilizing a photoelectric conversion engine to facilitate efficient bidirectional signal transmission. Through precise control of the transmission path and signal attenuation characteristics, the attenuation rate is maintained below 0.0035 dB/m at a wavelength of 850 nm, effectively ensuring stable and high-fidelity data transmission. The fiber-optic system is connected to the industrial-grade computing terminal located in the control room of the unloader via a USB 3.0 interface. The terminal is equipped with high-performance hardware, including an Intel Core i9-13900K processor (Intel, Santa Clara, CA, USA), an NVIDIA GeForce RTX 4060 GPU (Nvidia, Santa Clara, CA, USA), and 256 GB of DDR5 RAM.

## 4. Methodology

In railway bulk cargo unloading operations, the unloading hopper and the freight wagon serve as critical operational entities and measurement targets. Accurate and real-time acquisition of their relative spatial positions is essential for ensuring unloading efficiency and operational safety.

However, the unloading environment is often characterized by high reflectivity, low texture, and strong color similarity between targets, which presents severe challenges for visual perception systems in terms of feature interference and contour extraction, thereby hindering stable, continuous, and high-precision detection and localization.

Moreover, the compact deployment space, along with strong impact vibrations between the unloading hopper and bulk materials, degrades the stability of traditional GNSS RTK and LiDAR fusion-based solutions under such conditions. The inherent issues in multi-sensor systems—such as communication bandwidth bottlenecks, uncertain data transmission delays, and difficulties in temporal synchronization—further limit the real-time performance and localization accuracy of collaborative positioning frameworks.

**Figure 3:**
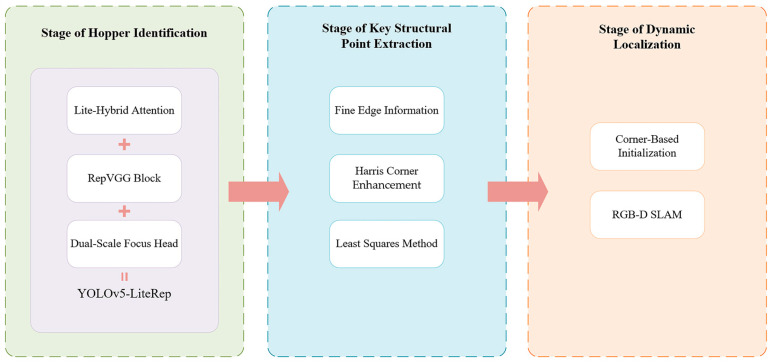
The overall system framework integrating three key modules: YOLOv5-based object detection, sub-pixel corner point extraction using image and depth information, and RGB-D SLAM-based dynamic 3D localization.

To address these challenges, we proposed a robust and high-precision localization method for railway bulk unloading hoppers based on a single-sensor architecture integrated with machine vision algorithms. The main method architecture is shown in Figure 3. This method also provides essential technical support for achieving automated unloading in complex operational scenarios.

### 4.1. Modeling of System Accuracy

The unloading operation of bulk materials in railway freight primarily involves two key components: the freight wagons and the bulk cargo unloading hoppers. To enable high-precision measurement of their relative spatial positions, large 3D cameras have been mounted on the four corners of the freight wagons to calibrate and continuously acquire the coordinates of critical structural points within a global world coordinate system. Simultaneously, a GNSS positioning module and a camera mounted with a nadir-viewing camera are installed on the upper end of the hopper’s mechanical arm to obtain the pose information of the hopper’s center point and to capture visual data throughout the automated unloading process.

However, due to limitations in camera resolution, image stability, and challenging environmental factors such as color uniformity and complex lighting conditions at the operation site, achieving accurate and real-time localization between the devices remains a significant challenge—especially in dynamic, dust-intensive bulk unloading environments.

Based on the calibrated parameters obtained from four 3D cameras, the three-dimensional poses of both the cargo hold and the unloading hopper can be reconstructed within the world coordinate system. The corner points of the cargo hold are designated as $O_{1}$, $O_{2}$,$O_{3}$,$O_{4}$, the actual geometric center of the unloading hopper is represented by $\left(x_{t},y_{t},z_{t}\right)$, whereas the predicted center obtained via the vision-based localization method is denoted as $\left(x_{p},y_{p},z_{p}\right)$.

The positioning accuracy is defined as a percentage by considering the spatial error along with the planar distance error between the unloading hopper and the freight wagon wall structure. The spatial error distance is defined as shown in Equation (1), which is used to evaluate the positional accuracy of the unloading hopper center point in the world coordinate system, calculated by the three-dimensional Euclidean distance formula:

(1) $$d_{s}=\sqrt{{\left(x_{p}-x_{t}\right)}^{2}+{\left(y_{p}-y_{t}\right)}^{2}+{\left(z_{p}-z_{t}\right)}^{2}}$$

Let the freight wagon wall be modeled as a plane π, with a normal vector n=(a,b,c). The plane equation is given by the following:

(2) π: ax+by+cz+d=0

The perpendicular distance from the camera-measured point $P_{pred}=(x_{p},y_{p},z_{p})$ to the wall plane is calculated as follows:

(3) $$d_{\pi }=\frac{ax_{p}+by_{p}+cz_{p}+d}{\sqrt{a^{2}+b^{2}+c^{2}}}$$

Taking the wall plane perpendicular to the *X*-axis as the reference for error evaluation, the wall-plane error of the unloading device is defined as $d_{\pi }$, the introduction of the maximum allowable wall error $D_{\pi }^{\max}$, precision P and $d_{\pi }$ inversely proportional to the relationship, the positioning accuracy is defined as being inversely proportional to the error—that is, the greater the error, the lower the accuracy. Accordingly, the accuracy metric is formulated as follows:

(4) $$P=\left\{\begin{array}{cc} \left(1-\frac{d_{\pi }}{x_{p}+d_{\pi }}\right)\cdot 100\%\; & d_{\pi }\leq D_{\pi }^{\max}\\ 0 & d_{\pi }\geq D_{\pi }^{\max} \end{array}\right.$$

Taking the Shijiazhuang railway bulk unloading yard as an example, the required average operational accuracy is 90%, with the maximum allowable wall-plane error $D_{\pi }^{\max}$ set to 0.60 m. Under current manual operations, the actual average maximum spatial error can reach up to 0.55 m. As defined in Equations (3) and (4), the predicted positioning accuracy can be calculated as follows:

(5) $$P=\left(1-\frac{0.55}{x_{p}+0.55}\right)\cdot 100\%\;$$

According to the calculation, the actual average operational accuracy of manual unloading is 93%. Therefore, this value is adopted as the minimum required benchmark for the predicted positioning accuracy of the proposed automated railway bulk unloading system, in order to ensure both operational precision and safety.

### 4.2. Stage of Hopper Identification

To achieve accurate localization of the unloading hopper in images, this study adopts an efficient one-stage object detection algorithm—YOLOv5 [23]. This method was originally proposed by Jocher et al., and possesses advantages such as fast detection speed and high accuracy, making it suitable for real-time object detection tasks in industrial scenarios. Although more recent versions such as YOLOv8, YOLOv9, and YOLOv11 have been proposed, YOLOv5 remains a preferred choice in this work due to its balanced performance, lower computational overhead, and modular structure. These characteristics make it easier to integrate with custom modules, such as the proposed LHA attention mechanism and lightweight RepVGG blocks, while maintaining compatibility with edge-device deployment constraints. These considerations make YOLOv5 a practical and extensible baseline for this application. The overall architecture of YOLOv5 consists of three key modules: the backbone, which extracts deep semantic features from images; the neck, which fuses multi-scale information to enhance the model’s ability to recognize objects of varying sizes; and the head, which outputs the final detection results, including object categories and location information. With this architecture, YOLOv5 can achieve fast and accurate recognition of the unloading hopper under complex backgrounds.

YOLOv5 offers five model versions with varying depths and widths, and their performance on the COCO dataset is shown in Table 2. Considering the resource constraints of edge computing devices, this study balances model complexity and deployment requirements, ultimately selecting YOLOv5l, which achieves a good trade-off between speed and accuracy, as the base architecture for detection.

#### 4.2.1. The Improved YOLOv5 Architecture

**Figure 4:**
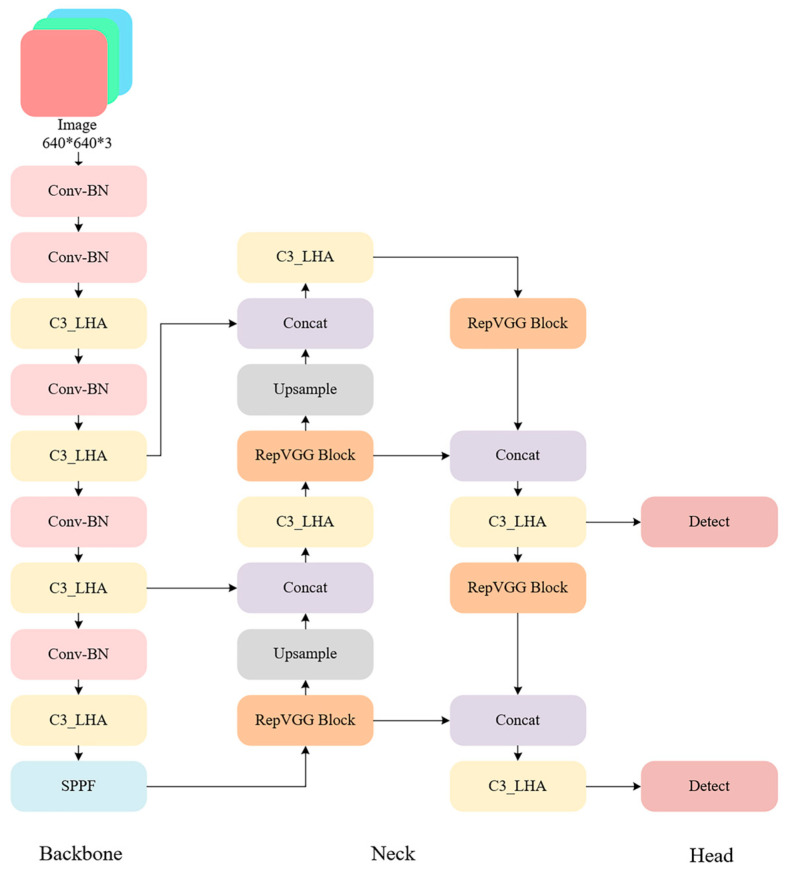
Architecture of the proposed YOLOv5-LiteRep, integrating the LHA-based backbone (Figure 4), RepVGG-enhanced neck, and a dual-scale detection head. This design improves both inference speed and localization accuracy for edge deployment.

Based on the module-level optimizations, this paper proposes systematic structural improvements to the YOLOv5 framework to accommodate the complex background interference, target scale variations, and embedded device resource constraints typical of batch offloading operations. The resulting model, called YOLOv5-LiteRep, is shown in Figure 4. The architecture consists of three main components. Each component is optimized to balance detection accuracy and inference efficiency through enhanced sensing, feature integration, and efficient detection design.

In the backbone, the original YOLOv5 C3 module is reconfigured with an LHA module to enhance network responsiveness to critical target regions. The LHA module combines ECA-Net, SimAM, and grouped convolutional strategies to effectively suppress background noise and highlight salient target features, while drastically reducing the number of parameters and computational cost. Embedding this attention module into multi-scale feature extraction can significantly improve the semantic awareness of the backbone system, especially in scenarios involving medium to large targets and occlusions.

In the neck, optimizations are twofold:

1. The standard convolution modules are completely replaced with RepVGG Blocks, which employ a multi-branch design during training to enhance nonlinear representation and leverage structural re-parameterization to fuse these branches into a single efficient 3 × 3 convolution during inference, significantly improving inference speed. This replacement not only boosts computational efficiency during feature fusion but also mitigates feature redundancy and separation problems commonly encountered in deep convolutional layers, thereby enhancing the model’s ability to generalize in complex scenarios.
2. The original C3 modules in the neck are substituted with the improved C3_LHA modules, facilitating full-path attention enhancement from shallow to deep layers and further strengthening the semantic consistency and discriminative power of deep features.

Regarding the head design, given the concentrated target scale distribution and fixed camera position in the application scenario, a scenario-adaptive dual-scale focused head (DSF-Head) is proposed. By removing the small-target detection branch and incorporating cross-layer feature reuse, the DSF-Head preserves fine recognition capabilities for medium and large targets. This design is especially well-suited for resource-constrained embedded deployments.

#### 4.2.2. YOLOv5 Backbone Network Enhanced by LHA

**Figure 5:**
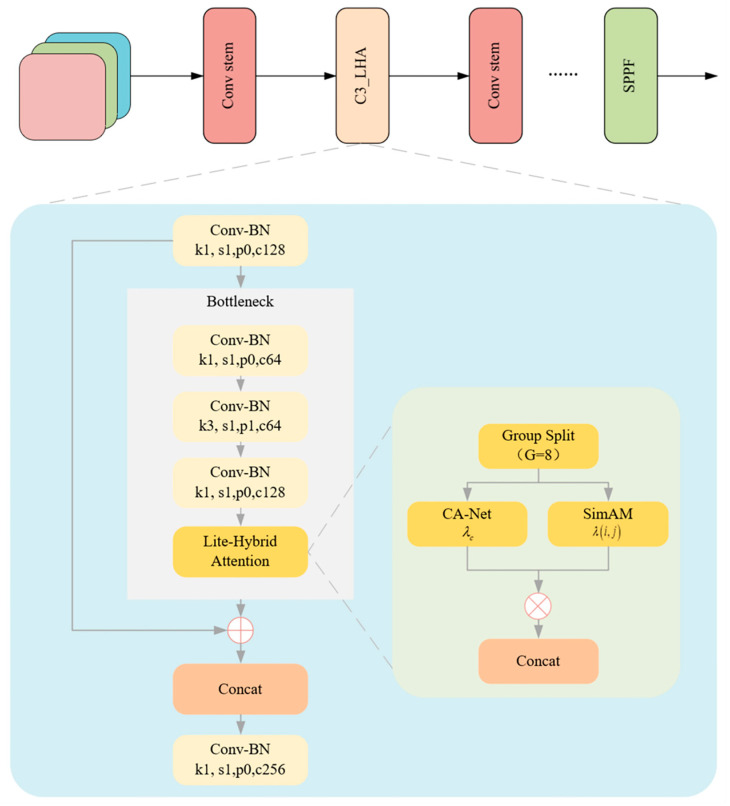
Detailed structure of the LHA-enhanced C3 module, which combines efficient channel attention (ECA-Net), SimAM spatial attention, and grouped convolutions to enhance feature sensitivity while maintaining computational efficiency.

To address the dual challenges of complex background interference in unloading operations and the limited computational resources of embedded systems, this paper proposes an embedded-friendly, lightweight hybrid attention module (LHA). The basic structure is illustrated in Figure 4. This module reconstructs the C3 basic unit in the YOLOv5 network by leveraging a multi-dimensional feature collaborative enhancement mechanism. It integrates efficient channel attention (ECA-Net) [24] and spatial attention (SimAM) [25], while employing grouped convolution to effectively control model complexity and significantly improve the network’s ability to perceive key target regions. Figure 5 presents the modified C3 module incorporating the proposed LHA mechanism. This component serves as a key element within the backbone of the improved YOLOv5-LiteRep architecture, as shown in Figure 5. Specifically, several C3 modules in the original backbone are replaced with the LHA-enhanced versions, contributing to improved attention and efficiency.

Considering the scale variation of targets in unloading scenes, the LHA module incorporates ECA-Net along the channel dimension to enable adaptive adjustment of feature responses. ECA replaces traditional fully connected operations with 1D convolution, significantly reducing computational and memory overhead, while capturing inter-channel dependencies through local interactions to enhance feature selectivity. Specifically, the input feature map $F\in R^{C\times H\times W}$ undergoes global average pooling across channels to generate a channel descriptor vector $V_{c}$, which is then passed through a 1D convolution layer followed by a sigmoid activation to output attention weights:

(6) $$\lambda_{c}=\sigma (\mathrm{ConvlD}(V_{c},k=3))$$

where $\lambda_{c}$ denotes the attention weights, σ is the sigmoid activation function, and the weights are applied to the original features through channel-wise multiplication to amplify responses of task-relevant channels.

While YOLOv5 utilizes residual connections and feature aggregation to provide implicit spatial modeling capabilities, these methods largely rely on deep stacked representations and lack explicit mechanisms to enhance responses at critical spatial locations. This limitation becomes apparent under heavy occlusion, strong background interference, or significant target scale variation. To address this, we introduce the SimAM module, an explicit spatial attention mechanism, to boost spatial focus.

SimAM requires no additional parameters and models neuron importance based on energy minimization principles. It evaluates the spatial significance of each location by calculating a local energy metric based on differences with neighboring features:

(7) $$E(i,j)=\sum_{\left(u,v)\in N(i,j\right)}{\left(x_{i,j}-x_{u,v}\right)}^{2}$$

where N(i,j) denotes the 3 × 3 neighborhood centered at (i,j), and a higher energy value indicates greater spatial distinctiveness. These energy scores are then converted into spatial attention weights via a sigmoid function:

(8) λ(i,j)=σ(−E(i,j))

To reduce the computational burden typically introduced by attention mechanisms, the proposed LHA module adopts a grouped convolution strategy along the channel dimension. Specifically, the input feature map is evenly divided into eight channel-wise sub-groups, each independently processed in parallel by ECA and SimAM spatial attention, and subsequently concatenated. This design significantly reduces redundancy and eliminates the need for global pooling or fully connected layers as used in CBAM.

Quantitatively, when applied to a standard feature map of size 80 × 80 × 256, the CBAM module requires approximately 8.92 million FLOPs, while the proposed LHA module only incurs 2.63 million FLOPs, representing a 70.5% reduction in computational overhead. The gain is primarily attributed to the lightweight nature of ECA (which uses local 1D convolutions instead of MLPs) and the grouping operation, which limits attention computation to lower-dimensional subspaces. This reduction in complexity leads to a measured inference latency improvement of 1.2 ms per frame in our test platform, without degrading detection accuracy.

#### 4.2.3. YOLOv5 Neck Network Enhanced by RepVGG Block

To enhance the representational capability and inference efficiency during the feature fusion stage, this paper introduces a structural re-parameterization convolution module—RepVGG Block [26]—into the neck component of YOLOv5.

The rationale for adopting RepVGG lies in its ability to achieve a favorable trade-off between accuracy and inference efficiency. During training, the RepVGG block employs a multi-branch architecture comprising standard 3 × 3 convolutions, 1 × 1 convolutions, and identity mappings. This multi-branch design enhances the non-linear modeling capability of the network. At inference time, all branches are mathematically merged into a single 3 × 3 convolution via structural re-parameterization, resulting in a simplified and hardware-friendly architecture without sacrificing learned representational capacity.

Compared to conventional depthwise separable convolutions and CSP-based designs commonly used in real-time detection frameworks, RepVGG preserves the dense connectivity of standard convolutions and avoids information loss caused by channel partitioning. These characteristics are especially advantageous in the context of hopper detection, where dense structural cues and multi-scale shape variations are prevalent.

When integrated into the neck of the proposed framework, the RepVGG block contributes to more coherent and robust feature fusion across scales. In conjunction with the C3_LHA modules in the backbone, this design enhances the model’s capacity to capture fine-grained spatial information while maintaining real-time performance, thereby improving detection reliability in complex industrial environments.

#### 4.2.4. Adaptive Detection Head Optimization

In the railway bulk unloading scenario, the distribution of target sizes under the camera view is relatively concentrated, and the need to detect small-scale objects is significantly reduced. To improve model efficiency and reduce redundant computation, this study introduces a scenario-specific modification to the YOLOv5 detection head. While the original YOLOv5 architecture employs a three-level hierarchical structure to detect small, medium, and large targets, our proposed dual-scale focused head (DSF-Head) retains only the medium and large target branches. The small-object detection layer is removed, and cross-scale feature interaction is preserved through a feature reuse mechanism.

**Figure 6:**
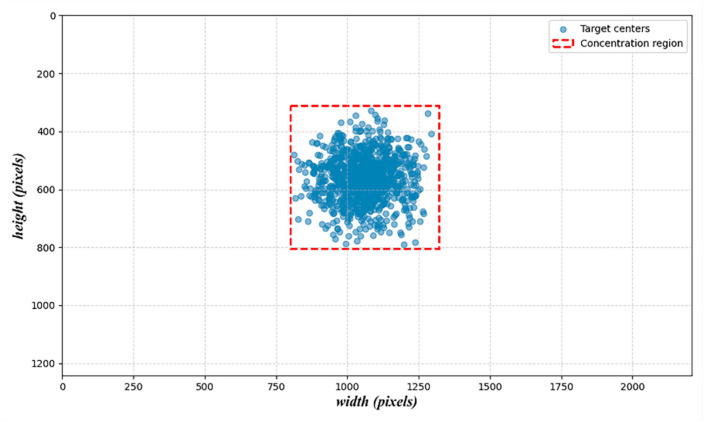
Target centers and concentration region for hopper detection scenario. The plot shows a spatial concentration of mid-sized targets within the camera view.

To provide empirical justification for this structural simplification, we conducted a simulation analysis based on visual patterns observed in our dataset. The objective was to quantitatively assess the spatial distribution and size characteristics of hopper targets under real-world operational conditions. As illustrated in Figure 6, most annotated targets are of moderate scale—with widths ranging from approximately 120 to 300 pixels and heights from 150 to 380 pixels—and exhibit a strong spatial clustering tendency. Notably, their centers are predominantly located within a fixed region of the image, bounded by 799–1320 pixels in width and 310–805 pixels in height.

This spatial regularity aligns with the geometric constraints of the unloading environment and the fixed placement of hoppers within the camera’s field of view. Consequently, the adoption of a dual-scale detection head is not only computationally efficient but also structurally justified. The simplified head design facilitates targeted optimization of anchor settings while maintaining detection accuracy for relevant objects in the scene.

### 4.3. Stage of Key Structural Point Extraction

**Figure 7:**
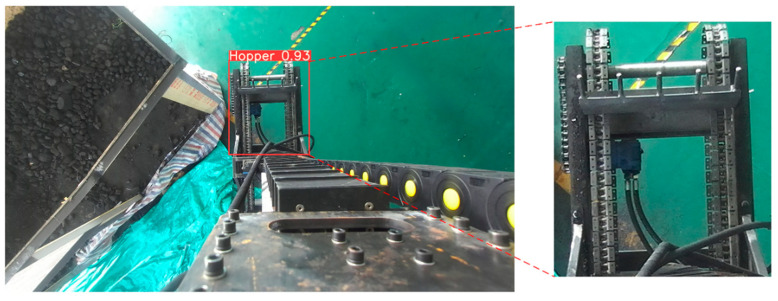
Identification diagram of the bulk unloading hopper.

After identifying the unloading hopper using the deep learning model, as illustrated in Figure 7, the task of precise localization becomes crucial. Due to the visual similarity in color between the unloading hopper, the freight wagon, and their background, traditional edge detection methods—which rely heavily on significant changes in color or brightness—often suffer from substantial missed detections in such scenarios. To address this, we propose a hybrid sub-pixel corner extraction strategy that combines RGB-based edge detection with depth-guided refinement to enhance accuracy and robustness. Unlike conventional approaches that rely solely on grayscale corner detectors, our method first applies a Scharr-filter-based Canny edge detector to improve boundary continuity, followed by polygonal contour simplification using the Douglas–Peucker algorithm.

Sub-pixel accuracy is further achieved by integrating the corner response function with depth map consistency, which enables the suppression of false positives and ambiguous corners. This strategy improves localization reliability under challenging industrial conditions, such as poor illumination, partial occlusion, and low-texture surfaces.

#### 4.3.1. Refined Edge Extraction

To accurately extract the 3D coordinates of key structural corners on the unloading hopper and freight wagon, we first ensure precise edge detection of the target structures in the image. This is achieved using the Canny edge detection algorithm, in which the traditional Sobel operator is replaced with the Scharr kernel during the gradient estimation phase to enhance edge responses and improve sensitivity to directional changes. This substitution enhances edge response strength and improves sensitivity to directional changes, offering significant advantages in scenarios with high color uniformity.

The Scharr kernel [27] is an optimized first-order derivative operator that increases the weighting of the central pixel in the convolution kernel, thereby enhancing edge response sensitivity and improving the stability of direction estimation. The convolution kernels used to extract horizontal and vertical gradients are defined as follows:

(9) $$G_{x}=\left[\begin{array}{ccc} -3 & 0 & +3\\ -10 & 0 & +10\\ -3 & 0 & +3 \end{array}\right],\;G_{y}=\left[\begin{array}{ccc} +3 & +10 & +3\\ 0 & 0 & 0\\ -3 & -10 & -3 \end{array}\right]$$

The image gradients in the horizontal and vertical directions can be expressed as follows:

(10) $$d_{x}=f(x,y)*G_{x},\;d_{y}=f(x,y)*G_{y}$$

where f(x,y) denotes the grayscale intensity of the input image, and ∗ represents the two-dimensional convolution operation. The overall gradient magnitude d and gradient direction θ at each pixel are then computed as follows:

(11) $$d=\sqrt{d_{x}^{2}+d_{y}^{2}},\;\theta =\arctan\left(\frac{d_{y}}{d_{x}}\right)$$

Subsequently, non-maximum suppression (NMS) and a dual-threshold hysteresis mechanism are introduced to further improve the accuracy and robustness of edge detection. NMS suppresses non-local maxima by retaining only the pixels with the highest gradient magnitude along the edge direction, thereby refining the edge structure. The dual-threshold mechanism classifies pixels based on a high threshold $T_{high}$ and a low threshold $T_{low}$: pixels with gradient magnitudes greater than $T_{high}$ are considered strong edges; those with values between $T_{low}$ and $T_{high}$ are treated as weak edges and retained only if they are connected to strong edges; pixels with gradient magnitudes below $T_{low}$ are discarded. This strategy effectively filters out noise and enhances the continuity and stability of the detected edges.

**Figure 8:**
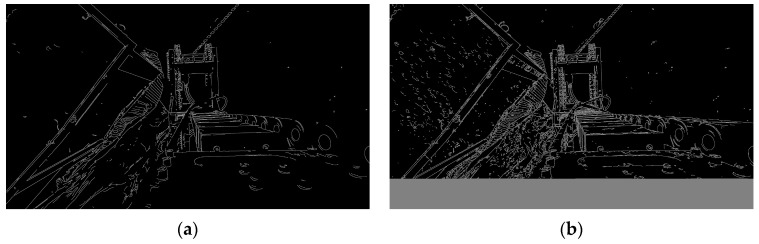
Diagram of edge detection: (**a**) Canny algorithm with Sobel operator; (**b**) Canny algorithm with Scharr kernel.

To determine the optimal parameter settings for typical operating conditions, experiments were conducted on a representative set of 100 image samples. The results show that under natural lighting conditions, edge detection results become significantly clearer and more continuous when $T_{low}$ is set to 136, and $T_{high}$ to 218, as illustrated in Figure 8.

However, edge features extracted using the Canny algorithm are highly susceptible to interference caused by color consistency and lighting variations in the scene. To further improve the accuracy and structural completeness of edge detection, this study introduces the DCC. The DCC utilizes the depth map as a visual representation of depth information to assist in edge validation and redundant feature elimination. Assume that the edge segment consists of n pixels, each with a corresponding depth value $D(x_{i},y_{i})$, and denote the average depth across the segment. The condition for depth consistency is defined as 0:

(12) $$\left|\frac{1}{n}\sum_{i=1}^{n}D(x_{i},y_{i})-D_{\mathrm{avg}}\right|\leq \Delta D_{\mathrm{threshold}}$$

where $\Delta D_{\mathrm{threshold}}$ is a predefined depth tolerance threshold.

Given that the unloading hopper and the freight wagon are independent structures with distinct spatial positions, this study adopts tailored edge extraction strategies for each. The unloading hopper features an irregular geometry and is consistently elevated above the bulk material. Therefore, within the YOLOv5 detection bounding box, a pixel-wise traversal filtering method is applied. Based on an extensive analysis of real-world image samples, we determine that setting $\Delta D_{\mathrm{threshold}}=0.10\;m$ enables complete and stable preservation of the fine edge features of the unloading hopper.

In contrast, the freight wagon exhibits pronounced linear edge characteristics. To exploit this structural regularity, additional edge segments are extracted from regions outside the YOLOv5 detection bounding box. After edge detection, candidate boundary segments are selected based on two constraints: (1) prominent linear shape, and (2) stable depth distribution. The edge pixels of qualified segments are then fitted using the least squares method (LSM) to generate precise linear models. Considering the geometric regularity and depth concentration of the freight wagon, $\Delta D_{\mathrm{threshold}}=0.03\;m$, which ensures effective and stable retention of its geometric boundaries.

**Figure 9:**
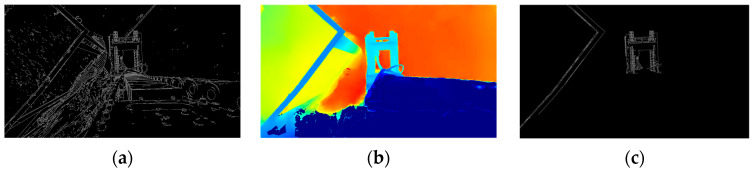
(**a**) Diagram of edge detection; (**b**) depth map; (**c**) target structure segmentation.

Experimental results, as illustrated in Figure 9, show that even with the application of depth consistency constraints, the unloading hopper—as a highly irregular 3D structure—still produces residual structural noise, resulting in blurred edge representations in the projection plane.

To achieve sub-pixel-level localization of the hopper’s boundaries, this study introduces a contour detection and polygonal approximation method. First, edge information is extracted from the grayscale image. Based on the object detection results from YOLOv5, A square binary mask (100 × 100 pixels) is centered within the YOLOv5 detection bounding box to constrain contour extraction to the core structural region of the hopper. This targeted masking strategy reduces peripheral noise interference and enhances the reliability of sub-pixel edge detection.

**Figure 10:**
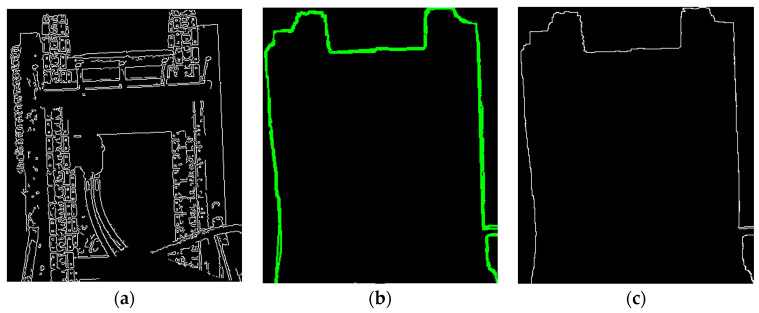
(**a**) Edge map within YOLOv5 bounding boxes; (**b**) Douglas–Peucker algorithm and mask-based edge refinement.; (**c**) refined edge extraction results.

Then, external contours are extracted within the mask, and the Douglas–Peucker algorithm is applied to approximate the edge curves with simplified and smoothed polygonal representations. This process enhances the geometric stability of the edges and reduces local noise. The proposed method is implemented on the OpenCV library [28] and achieves sub-pixel-level edge extraction while preserving essential geometric features, as illustrated in Figure 10.

#### 4.3.2. Harris Operator for Corner Point Enhancement

After the primary edge structures of both the unloading hopper and the freight wagon are extracted with reasonable accuracy. To further enhance the stability and robustness of key corner point identification—particularly under challenging conditions involving frequent dynamic changes and low image contrast—this study introduces a Harris operator-based corner enhancement method, combined with geometric consistency constraints for candidate selection and refinement.

The Harris operator is a corner response function based on variations in image intensity gradients. It generates strong responses at edge intersections or regions of significant local intensity variation, making it widely used for the precise localization of structural corners. In this study, Harris corner response calculations are limited to image regions that have already passed edge detection and depth filtering, thereby significantly reducing the risk of false positives caused by background clutter and improving both detection accuracy and computational efficiency. The response function R is defined as follows:

(13) $$R=\det(M)-k\cdot {\left(\mathrm{trace}(M)\right)}^{2}$$

where M represents the auto-correlation matrix of image intensity gradients within a local window; det(M) and $\mathrm{trace}\left(M\right)$ denote its determinant and trace, respectively; and k is an empirical constant. A pixel is considered a valid corner point if its response value R exceeds a predefined threshold.

To select corner points that better conform to the geometric characteristics of the target structures from the initial Harris response point set, this paper introduces two geometric consistency constraints:

1. Near-Orthogonality Constraint:

For each candidate corner, two sets of edge pixels are extracted along the principal directions of the intersecting edges within its local neighborhood. Each edge segment is fitted using the least squares method (LSM) to obtain its principal direction vector, denoted as $\vec{v_{1}}$ and $\vec{v_{2}}$. The cosine of the angle between the two direction vectors is computed as follows:

(14) $$\cos\theta =\frac{\vec{v_{1}}\cdot \vec{v_{2}}}{\left\|\vec{v_{1}}\right\|\cdot \left\|\vec{v_{2}}\right\|}$$

If the following condition is satisfied:

(15) $$\left|\cos\theta \right|\leq \tau$$

If the two edges are approximately orthogonal, as determined by a cosine threshold τ empirically set to 0.2, the corner with the smallest deviation from the ideal right angle (90°) is selected as the final structural key point.

2. Edge Continuity Constraint:

To ensure that each corner point lies at the intersection of two strongly responsive and continuous edge segments, the local gradient magnitude is evaluated using a sliding average method. Specifically, two 21 × 21 pixel windows—centered on the corner point and aligned with each edge direction—are defined as $W_{1}$ and $W_{2}$. The average gradient magnitudes within these windows are calculated as follows:

(16) $$G_{i}=\frac{1}{\left|W_{i}\right|}\sum_{p\in W_{i}}\nabla I(p),\;i=1,2$$

**Figure 11:**
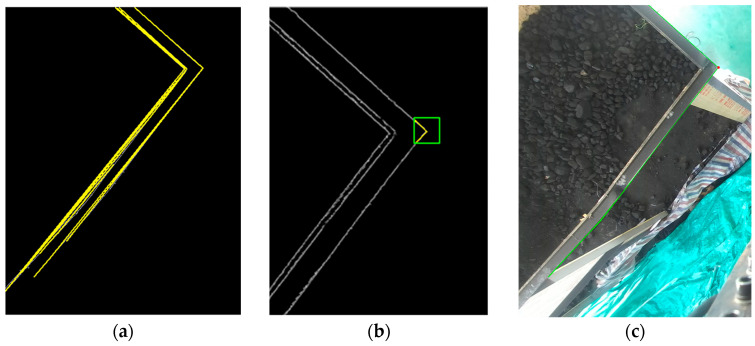
(**a**) Plot of linear fit using LSM; (**b**) result of corner point extraction; (**c**) extraction results of freight hold edges and corner point.

**Figure 12:**
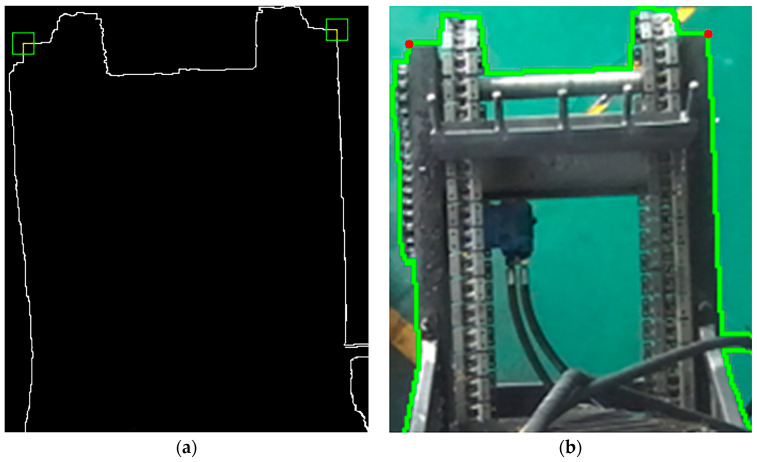
(**a**) Result of corner point extraction; (**b**) extraction results of unloading hopper edges and corner point.

If both $G_{1}$ and $G_{2}$ exceed a predefined threshold $G_{min}=0.3$, the local edge responses are considered strong and continuous, and the corner point is retained as a key point. Otherwise, it is discarded. Figure 11 illustrates the extracted edges of the freight hold along with their corresponding corner points, while Figure 12 presents the identified corner points and refined edge contours of the unloading hopper.

### 4.4. Stage of Dynamic Localization

Due to the inability to perform full-view measurements of the freight wagon under real operating conditions, the system is constrained by a limited field of view. As a result, the PnP-based pose estimation model is prone to failure when key feature points are occluded or missing during the motion of the unloading hopper. To address this issue, this paper proposes a hybrid pose estimation strategy that combines corner point-based initialization with RGB-D SLAM [29,30] tracking, enabling stable and continuous localization of the hopper target.

At system initialization, only a single visible corner point and its neighboring edge direction—extracted via image processing algorithms—are used, together with prior structural knowledge of the freight wagon, to construct the initial world coordinate reference frame. This reference frame provides an accurate initialization for subsequent pose tracking using RGB-D SLAM, thereby significantly enhancing localization robustness and stability under constrained visual conditions.

**Figure 13:**
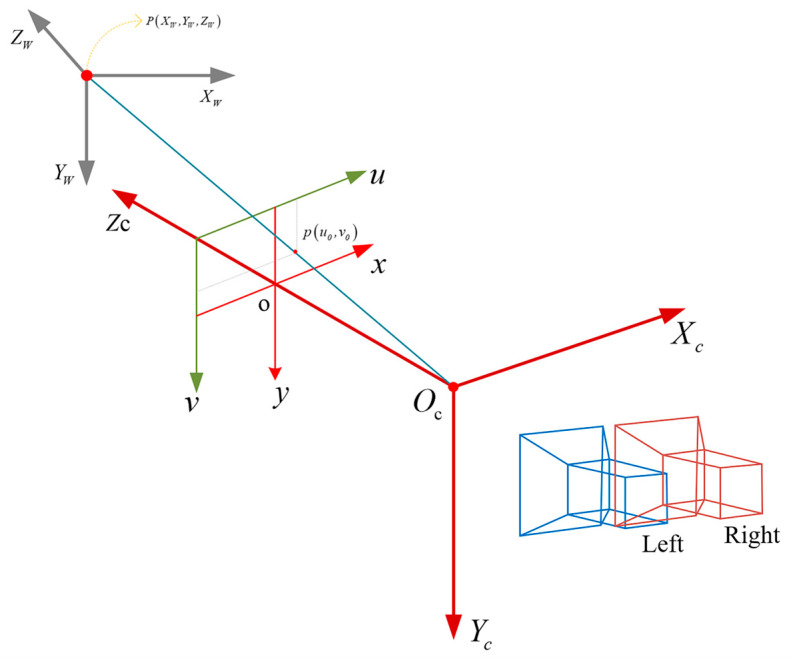
Principle of stereo vision-based 3D measurement, illustrating how pixel coordinates and depth values are transformed into 3D world coordinates using intrinsic camera parameters and back-projection.

Firstly, the principle of 3D measurement based on stereo vision is illustrated in Figure 13. Based on this principle, the depth value $d_{0}$, which is obtained from the depth map, and the camera intrinsic matrix K, are used to back-project the pixel coordinates $\left(u_{0},v_{0}\right)$ of the detected corner point into the camera coordinate system, yielding its 3D position. This process transforms image coordinates into the camera coordinate system using the relation:

(17) $$P_{C}=d_{0}\cdot K^{-1}\left[\begin{array}{c} u_{0}\\ v_{0}\\ 1 \end{array}\right]=\left[\begin{array}{c} X_{C}\\ Y_{C}\\ Z_{C} \end{array}\right]$$

Next, leveraging the two structural edge segments of the freight wagon extracted by the prior image processing algorithm, their corresponding pixel direction vectors ${\vec{v}}_{x}^{\left(2D\right)},{\vec{v}}_{y}^{\left(2D\right)}$ in the image plane are obtained. These vectors are subsequently back-projected into three-dimensional space, resulting in unit direction vectors ${\vec{X}}_{C},{\vec{Y}}_{C}$ expressed in the spatial coordinate system:

(18) $${\vec{X}}_{C}=\frac{P_{C}^{x_{2}}-P_{C}^{x_{1}}}{∥P_{C}^{x_{2}}-P_{C}^{x_{1}}∥},\;{\vec{Y}}_{C}=\frac{P_{C}^{y_{2}}-P_{C}^{y_{1}}}{∥P_{C}^{y_{2}}-P_{C}^{y_{1}}∥}$$

Next, these two unit direction vectors are used to compute the surface normal vector ${\vec{Z}}_{C}={\vec{X}}_{C}\times {\vec{Y}}_{C}$, thereby defining a local world coordinate system whose origin is the visible corner point of the freight wagon and whose axes are aligned with the edge directions. This coordinate transformation can be expressed as follows:

(19) $$T_{W\to C_{0}}=\left[\begin{array}{cccc} {\vec{X}}_{C} & {\vec{Y}}_{C} & {\vec{Z}}_{C} & P_{C}\\ 0 & 0 & 0 & 1 \end{array}\right]$$

where W represents the camera coordinate system, and $T_{W\to C_{0}}$ denotes the transformation from the camera to the world coordinate system.

After constructing the local world coordinate system based on the initial corner point, the system enters the continuous operation phase of the unloading hopper. Due to dynamic changes in the camera’s viewpoint, the static corner point can no longer be reliably observed. As a result, the task of pose estimation is handed over to the RGB-D SLAM module. This module tracks depth information and visual feature points across image frames to continuously estimate the camera’s pose relative to the initial frame in real time:

(20) $$T_{C_{0}\to C_{t}}=\left[\begin{array}{cc} R_{t} & t_{t}\\ 0 & 1 \end{array}\right]$$

where $R_{t}$ denotes the rotation matrix and $t_{t}={\left[x_{t},y_{t},z_{t}\right]}^{T}$ represents the translation vector of the current frame.

By left-multiplying the relative pose transformation matrix output by the RGB-D SLAM module with the initial camera pose in the world coordinate system, the real-time pose of the current frame in the world coordinate system can be computed as follows:

(21) $$T_{W\to C_{t}}=T_{W\to C_{0}}\cdot T_{C_{0}\to C_{t}}$$

Considering the rigid connection between the camera and the unloading hopper, the position of the hopper’s corner point in the camera coordinate system is measurable in real time. Therefore, its real-time position in the world coordinate system can be expressed as follows:

(22) $$P_{W,t}=T_{W\to C_{t}}\cdot P_{C}^{\left(0\right)}=\left[\begin{array}{cc} R_{W} & t_{W}\\ 0 & 1 \end{array}\right]\cdot \left[\begin{array}{c} X_{C}^{\left(0\right)}\\ Y_{C}^{\left(0\right)}\\ Z_{C}^{\left(0\right)}\\ 1 \end{array}\right]=\left[\begin{array}{c} X_{W}^{\left(t\right)}\\ Y_{W}^{\left(t\right)}\\ Z_{W}^{\left(t\right)}\\ 1 \end{array}\right]$$

where $P_{C}^{\left(0\right)}$ denotes the measured position of the hopper’s corner points in the camera coordinate system at system initialization, as defined in Equation (17), which remains constant; $\left(X_{W}^{\left(t\right)},Y_{W}^{\left(t\right)},Z_{W}^{\left(t\right)}\right)$ represents the 3D positions of the hopper’s corner points at frame t.

## 5. Experiment and Evaluation

### 5.1. Experimental Environment

The experimental system is built on the Ubuntu 22.04 operating system. The software environment is based on Python 3.8.20, with PyTorch 2.4.1 serving as the deep learning framework, in conjunction with CUDA 12.4 and torchvision 0.19.1. During model training, all input image sizes are uniformly set to 1280 × 1280, with a total of 200 training epochs and a batch size of 16. The ADAM optimizer is used to ensure training stability while accelerating convergence and improving final accuracy.

### 5.2. Dataset Construction

As the “crawler-style” unloading hopper is a self-developed piece of equipment, there are no publicly available image datasets for relevant operational scenarios. Therefore, a custom dataset was constructed for this study. Images were captured using a downward-facing camera (ColorVu 2.0, Hangzhou Hikvision Digital Technology Co., Ltd., Hangzhou, China) mounted at the end of a robotic arm. A total of 800 images were initially collected, covering a range of spatial positions, orientation angles, and lighting conditions. All images were preprocessed and uniformly resized to 2208 × 1242 (2K resolution). Precise manual annotations were performed using the LabelImg tool, with all labels standardized as “Hopper”, ensuring that the key structural components of the unloading hopper were fully enclosed within the detection boxes.

**Figure 14:**
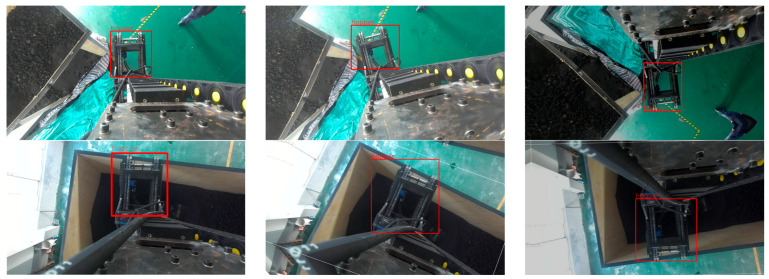
Sample images from the hopper dataset.

As shown in Figure 14, to improve the model’s robustness and generalization across diverse working conditions, data augmentation was applied using the open-source image processing libraries Albumentations and OpenCV. Techniques such as brightness adjustment, horizontal flipping, shifting, rotation, and scaling were used to significantly increase the diversity and complexity of the dataset. Sample images are shown in Figure 14; the final expanded dataset contains approximately 2200 images.

### 5.3. Experimental Results

To evaluate the effectiveness of the proposed improvements in the hopper recognition phase, we conducted both comparative and ablation experiments based on the YOLOv5 baseline model. As shown in Table 3, the baseline YOLOv5 achieves a mean average precision (mAP) of 86.1% on the hopper detection dataset.

To further investigate the individual contributions of the proposed architectural enhancements, we performed a series of ablation studies focusing on the lite-hybrid attention (LHA) module integrated into the backbone and the RepVGG block applied to the neck. Introducing the LHA module alone increases the mAP to 87.9%, demonstrating the effectiveness of attention mechanisms in enhancing spatial and channel-level feature representations. When the RepVGG block is employed independently in the neck, the mAP improves to 87.2%, along with a 0.7 ms reduction in inference latency due to structural re-parameterization.

The combined configuration, incorporating both LHA and RepVGG, yields the best performance, achieving an mAP of 88.6%. These results confirm the complementary nature of the two modules: LHA enhances contextual feature encoding, while RepVGG facilitates efficient multi-scale feature fusion with minimal computational overhead. The ablation study validates that the proposed modules jointly contribute to both detection accuracy and inference efficiency in the hopper recognition scenario.

In addition, we evaluated the proposed YOLOv5-LiteRep model on the COCO dataset to examine its applicability in general object detection tasks. As shown in Table 4, our model achieved a mAP@0.5 of 69.2%, surpassing the baseline YOLOv5l (67.3%). This result indicates that the proposed architectural improvements are not only effective in domain-specific scenarios such as hopper detection, but also generalize well to complex public datasets with diverse object categories and scales.

**Figure 15:**
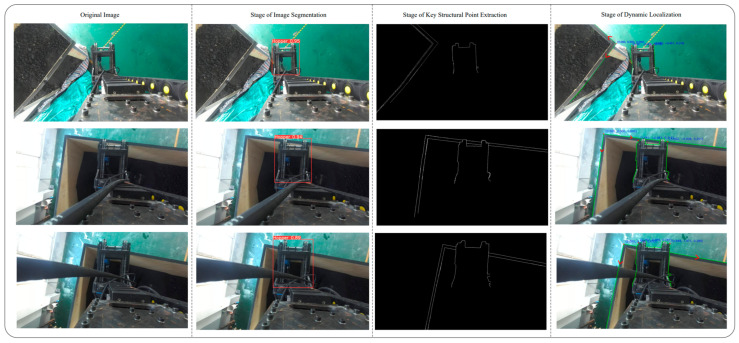
Three-stage experimental procedure for identification and localization.

We selected three corner points located at different positions to initiate the corner point-based initialization and localization experiment, as illustrated in Figure 15. It is worth noting that experimental uncertainty is inevitable in scientific research. During the experiment, a latch unexpectedly detached, causing a hydraulic hose to enter the camera’s field of view and introduce significant spatial interference. To better evaluate the performance of the proposed method in complex real-world scenarios, we deliberately retained this interference in the dataset as part of the scientific validation process, thereby enabling a more robust assessment of the method’s practical applicability.

To evaluate the localization accuracy of the proposed method, we selected the spatial coordinates of the front-left corner of the unloading hopper as the target for relative error analysis. Since the hopper and the freight wagon are standardized industrial components with flat surfaces and well-defined corner structures, their physical geometry serves as a natural spatial reference for localization. The ground truth positions of these corner points were obtained through manual measurement using calibrated laser ranging tools. This setup allows for objective and reproducible assessment of localization performance without the need for additional artificial markers or reference structures. The localization error is calculated according to Equation (5). We conducted 30 positioning trials within the freight wagon to record the time-varying coordinates of the selected point, resulting in 30 sets of spatial point data, as shown in Table 5.

**Figure 16:**
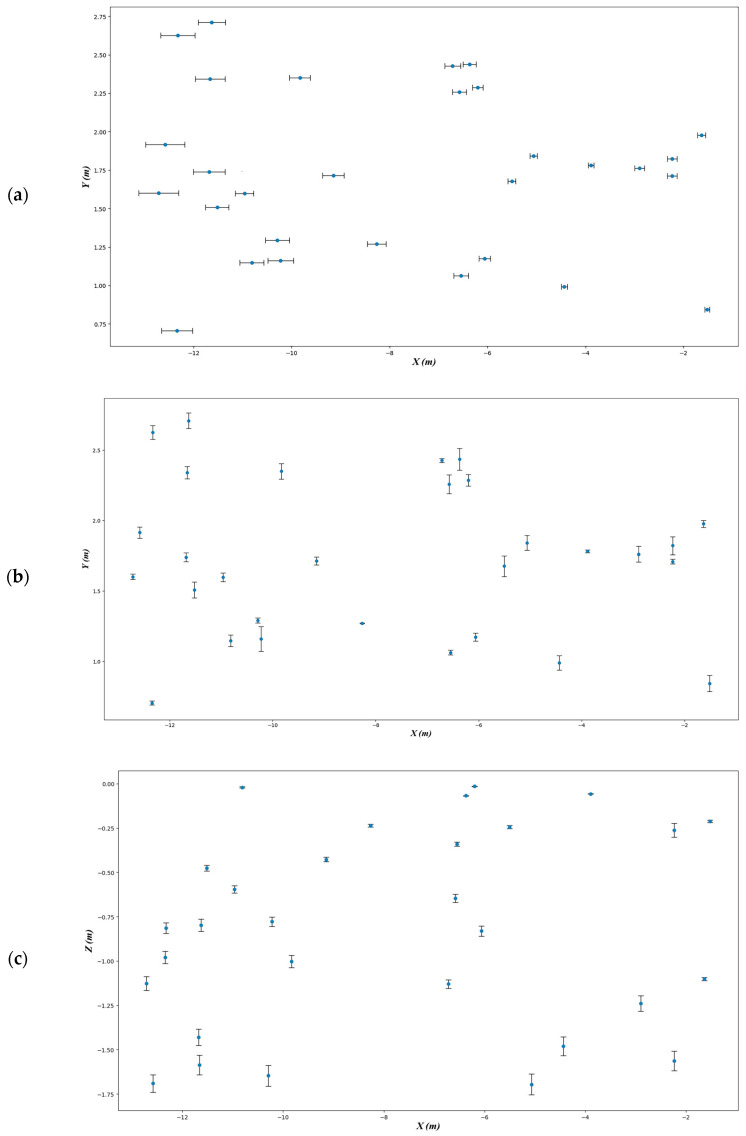
Visualization of 3D localization accuracy over 30 trials: (**a**) *X*-axis error distribution, (**b**) *Y*-axis error distribution, and (**c**) *Z*-axis error distribution, confirming the system’s robustness and precision under realistic conditions.

We further quantified the relative errors from these trials and visualized the results in the error analysis plot (Figure 16), which provides an intuitive assessment of the localization accuracy and the consistency of the proposed method across repeated measurements.

## 6. Conclusions and Future Work

In this study, a vision-based localization framework employing only one visual sensor is proposed to achieve real-time detection and 3D positioning of railway bulk unloading hoppers. The proposed approach leverages machine vision techniques and integrates an improved YOLOv5 model with image processing methods to achieve fast and accurate detection of the unloading hopper in complex scenes. By incorporating DCC and geometric constraints, sub-pixel-level corner point coordinates are extracted. To address pose estimation failures caused by a limited field of view in PnP algorithms, a corner-based initialization strategy is designed and combined with RGB-D SLAM to achieve dynamic, high-precision localization of the unloading hopper.

To validate the effectiveness of the method, 30 positioning experiments were conducted. Results show that the proposed approach achieves an average spatial localization error of 0.202 m, with a mean positioning accuracy of 97.07%. Compared to manual operations with an average accuracy of 93%, the proposed approach achieves a 4.07% improvement in positioning accuracy, effectively advancing both automation and operational efficiency in bulk unloading tasks. Moreover, the use of a single-sensor configuration offers notable advantages in terms of deployment simplicity and cost-effectiveness. Compared to traditional multi-sensor systems, the proposed solution reduces the number of sensors by approximately 83%, and overall equipment costs by around 90%, demonstrating strong potential for practical deployment and scalability.

Despite the promising results, there is still room for improvement in terms of system robustness. The complex environmental conditions of bulk cargo unloading sites—particularly dust contamination—can degrade visual sensor performance and affect recognition stability. In addition, vibrations from the robotic arm inevitably impact localization accuracy. Future research will focus on enhancing system stability and anti-interference capability under harsh operating conditions. Furthermore, deeper integration between visual perception and automated control systems will be explored to enable more accurate, faster, and more stable autonomous unloading operations.

## Figures and Tables

**Table 1:** Camera technical specifications.

Focal Length	2.1 mm (Fixed Focus)
Field of view	110° (H) × 70° (V) × 120° (D)
Aperture	F/1.8
IMU	3-axis gyro + accel

**Table 2:** Performance of YOLOv5 model on the COCO dataset.

Model	mAP@0.5 (%)	Speed (ms)	FLOPs (G)	Params (M)
YOLOv5n	45.7	45	4.5	1.9
YOLOv5s	56.8	98	16.5	7.2
YOLOv5m	64.1	224	49.0	21.2
YOLOv5l	67.3	438	109.1	46.5
YOLOv5x	68.9	785	212.0	86.7

**Table 3:** Ablation results for LHA and RepVGG modules based on YOLOv5L.

Model	Params (M)	mAP (%)	FLOPs (G)	Latency (ms)
YOLOV5L	46.5	86.1	116.4	9.5
+ LHA (Backbone only)	47.7	87.9	121.3	9.8
+ RepVGG (Neck only)	49.5	87.2	114.2	8.8
+ LHA + RepVGG (Ours)	50.9	88.6	119.6	8.6

**Table 4:** Comparative performance of YOLOv5-LiteRep and YOLOv5l on the COCO dataset.

Model	Params (M)	mAP (%)	FLOPs (G)	Latency (ms)
YOLOV5L	46.5	67.3	109.1	9.5
YOLOv5-LiteRep	50.9	69.2	112.4	8.9

**Table 5:** Spatial coordinate results of 30 positioning trials for the front-left corner of the unloading hopper.

Serial Number	Predicted Points	*X*-Axis Offset Distance (m)	*Y*-Axis Offset Distance (m)	*Z*-Axis Offset Distance (m)	Spatial Offset Distance (m)
1	(−1.518, 0.844, −0.212)	0.053	0.058	0.007	0.088
2	(−1.633, 1.977, −1.102)	0.086	0.024	0.009	0.092
3	(−2.231, 1.823, −1.563)	0.101	0.063	0.055	0.142
4	(−2.236, 1.711, −0.263)	0.101	0.017	0.039	0.109
5	(−2.896, 1.762, −1.239)	0.099	0.056	0.043	0.116
6	(−3.889, 1.782, −0.057)	0.056	0.010	0.002	0.057
7	(−4.434, 0.991, −1.481)	0.066	0.051	0.052	0.097
8	(−5.065, 1.842, −1.696)	0.074	0.053	0.059	0.104
9	(−5.504, 1.678, −0.245)	0.079	0.073	0.009	0.108
10	(−6.058, 1.174, −0.831)	0.117	0.029	0.029	0.125
11	(−6.202, 2.288, −0.015)	0.111	0.041	0.001	0.119
12	(−6.370, 2.437, −0.068)	0.131	0.078	0.002	0.154
13	(−6.544, 1.064, −0.341)	0.152	0.016	0.012	0.154
14	(−6.576, 2.259, −0.647)	0.142	0.066	0.023	0.157
15	(−6.714, 2.428, −1.130)	0.165	0.014	0.025	0.169
16	(−8.265, 1.271, −0.237)	0.189	0.003	0.008	0.190
17	(−9.149, 1.715, −0.427)	0.222	0.029	0.012	0.226
18	(−9.831, 2.351, −1.003)	0.212	0.056	0.035	0.222
19	(−10.226, 1.161, −0.778)	0.262	0.087	0.027	0.278
20	(−10.293, 1.293, −1.647)	0.246	0.018	0.058	0.254
21	(−10.815, 1.148, −0.020)	0.243	0.041	0.003	0.249
22	(−10.963, 1.599, −0.596)	0.185	0.031	0.021	0.189
23	(−11.521, 1.509, −0.476)	0.236	0.056	0.017	0.243
24	(−11.632, 2.711, −0.798)	0.278	0.056	0.035	0.283
25	(−11.665, 2.342, −1.586)	0.303	0.044	0.055	0.310
26	(−11.683, 1.740, −1.430)	0.326	0.031	0.046	0.331
27	(−12.327, 2.627, −0.814)	0.351	0.049	0.029	0.357
28	(−12.342, 0.705, −0.979)	0.319	0.014	0.034	0.322
29	(−12.584, 1.915, −1.691)	0.397	0.039	0.049	0.403
30	(−12.714, 1.602, −1.126)	0.408	0.019	0.039	0.412
Positioning Accuracy P (%)		97.35	97.54	96.32	97.07

## Data Availability

Part of the data used in this study (i.e., the COCO dataset) is publicly available at https://cocodataset.org.
